# Economic burden due to COVID-19 in a Colombian Caribbean state, 2020 and 2021

**DOI:** 10.1590/S2237-96222024V33E2023830.EN

**Published:** 2024-07-08

**Authors:** Fernando Salcedo-Mejía, Lina Moyano-Tamara, Josefina Zakzuk, Ana Milena Lozano, Héctor Serrano-Coll, Bertha Gastelbondo, Salim Mattar Velilla, Nelson Rafael Alvis Zakzuk, Nelson J. Alvis-Zakzuk, Nelson Alvis Guzmán

**Affiliations:** 1 ALZAK Foundation, Cartagena, Bolívar, Colômbia; 2 Universidad de Cartagena, Grupo de Investigación en Economía de la Salud, Cartagena, Bolívar, Colômbia; 3 Universidad CES, Instituto colombiano de Medicina tropical, Apartadó, Colômbia; 4 Universidad de Córdoba, Instituto de Investigaciones Biológicas del Trópico, Montería, Córdoba, Colômbia; 5 Universidad de la Costa, Departamento de Ciencias de la Salud, Barranquilla, Atlántico Colômbia

**Keywords:** Health Care Costs, Cost of Illness, Economic Burden, COVID-19, Costos de la Atención en Salud, Costo de Enfermedad, Carga Económica, COVID-19

## Abstract

**Objective:**

To estimate the economic burden associated with COVID-19 in Córdoba, Colombia, 2020 and 2021.

**Methods:**

Economic burden study. Direct costs were analyzed from the third-party payer perspective using healthcare administrative databases and interviews from a cohort of confirmed COVID-19 cases from Córdoba. Costing aggregation was performed by the bottom-up method. Indirect costs were estimated using the productivity loss approach. Contrast tests and statistical models were estimated at 5% significance.

**Results:**

We studied 1,800 COVID-19 cases. The average economic cost of COVID-19 per episode was estimated at US$ 2,519 (95%CI 1,980;3,047). The direct medical cost component accounted for 92.9% of the total; out-of-pocket and indirect costs accounted for 2% and 5.1%, respectively.

**Conclusion:**

COVID-19 economic cost was mainly due to direct medical costs. This study provided evidence of the economic burden faced by households due to COVID-19, with the most vulnerable households bearing much of the burden on their income.

## Study contributions

**Main results:**

The mean economic cost of COVID-19 per episode was estimated in US$ 2,519 (95%CI 1,980;3,047). The direct medical cost component accounted for 92.9% of the total; direct expenses and indirect costs accounted for, 2% and 5.1%, respectively.

**Implications for services:**

Estimating the costs of a disease is necessary for any healthcare system, especially its own expenses and indirect costs. These are the main inputs for evaluating the cost-effectiveness of new health interventions.

**Perspectives:**

In future analyses it will be important to estimate the subsequent costs due to impairments caused by long COVID-19, in order to complete the estimate of the economic burden of this disease.

## Resumen

**Objetivo:**

Estimar la carga económica asociada al COVID-19 en Córdoba, Colombia, entre 2020 y 2021.

**Métodos:**

Estudio de carga económica. Los costos directos se analizaron desde la perspectiva del tercer pagador usando bases de datos administrativas y entrevistas. La agregación del costeo se realizó por el método de bottom-up. Los costos indirectos se estimaron usando el enfoque de pérdida de productividad. Los test de contraste y modelos estadísticos se estimaron al 5% de significancia.

**Resultados:**

Se analizaron 1.800 casos de COVID-19. El costo económico promedio por episodio fue US$ 2.519 (IC_95%_ 1.980;3.047). Los costos directos médicos explicaron el 92,9% del total; el gasto de bolsillo y los costos indirectos un 2% y 5,1%, respectivamente.

**Conclusión:**

El costo económico del COVID-19 fue principalmente por el costo directo médico. Este estudio evidenció la carga económica que enfrentan los hogares debido al COVID-19, siendo los más vulnerables quienes asumieron la mayor parte en sus ingresos.

## INTRODUCTION

Between December 2019, when COVID-19 was identified, and mid-April 2021, there were more than 411 million cases worldwide and approximately 2.9 million deaths.^1^ In Colombia, as of April 14, 2021, 2,585,801 cases and 66,819 deaths from COVID-19 had been reported, and while some cities were already experiencing their third peak, others were in their second.^2^ Regional differences in incidence and mortality rates and case fatality ratios were also recorded.^2^ This fact, associated with the specificities of each region, makes each of the country’s Departments unique, with regard to the impact of the pandemic.

The effects of COVID-19 on health systems have had an impact on the ability to address the pandemic and, at the same time, maintain the supply of these services.^3^ Such effects appear to be even greater in low- and middle-income countries, with significant health costs. It has been estimated that the costs of responding to COVID-19, in a four-week scenario, were approximately US$ 52 billion, at a rate of US$ 8.60 *per capita*.^4^ In turn, families faced not only unexpected job and income losses during the pandemic, but also medical expenses to care for members who contracted the disease. These direct expenses further affected their reduced income, worldwide, in the context of the pandemic.^5^ ^),(^ ^6^

Estimating the economic burden of a disease provides information on direct medical costs, non-medical costs (out-of-pocket expenses) and indirect costs associated with its management. Since cost analysis approaches for COVID-19 in Colombia are still scarce, it is valid to economically evaluate the frequency of their use by health services and, especially, to gain knowledge of the direct expenses and costs associated with lost productivity, which, are usually not estimated in studies of the cost of an illness. For this reason, the objective of this study was to estimate the economic burden associated with COVID-19 in Córdoba, Colombia, between 2020 and 2021.

## METHODS

*Study design*

This is an evaluation study of the economic burden of COVID-19, from the perspective of society, between March 2020 and April 2021.

*Study location and background*

Córdoba is a Department located in the north of Colombia with high COVID-19 mortality. In 2020, it consisted of 30 municipalities and 1,838,574 inhabitants.^7^ The Department’s capital, Montería, was one of the most affected cities at the beginning of the pandemic. On April 14, 2021, it recorded one of the lowest cumulative incidence rates among the main cities in the Colombian Caribbean, but its mortality rate was the highest in the region.^2^

*Participants*

**Supplementary Figure 1:**
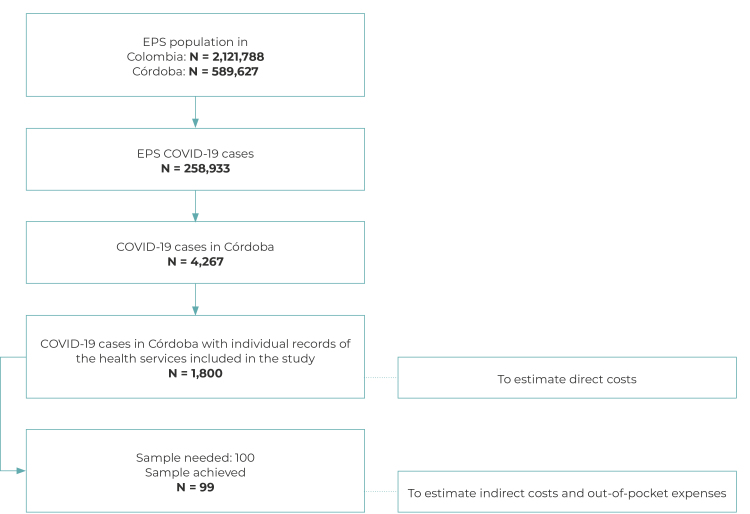
‒ Sample selection process for estimating out-of-pocket expenses and indirect costs, Córdoba, Colombia, between 2020 and 2021

Diagnosed COVID-19 cases treated by the health system in Córdoba, Colombia. Supplementary Figure 1 presents the flowchart for selecting COVID-19 cases for economic cost estimates. Of the total COVID-19 cases in Córdoba, those for which frequencies were available regarding use of health services were analyzed for direct medical costs and a subsample was selected to estimate out-of-pocket expenses and indirect costs.

*Data source and variables*

*Direct medical cost data*

We analyzed health service administrative databases based on a cohort of positive COVID-19 cases in Córdoba. The Empresa Promotora de Salud (EPS), which generated these databases, identified 4,267 cases of COVID-19 in the Department by the end of 2020 (Supplementary Figure 1). Its subscribers, who belong to both health care types existing under the Colombian health system [contributory (subscribers who are workers or people with the ability to pay) and subsidized (subscribers who are people with low income)], accounted for 30.2% of the Department’s population in 2020. Information was then collected on symptoms, severity and comorbidities, services and costs in outpatient services, home care and hospitalization in clinics and hospitals in the Department.

*Variables for direct medical costs*

The available variables were demographic: sex (female, male), age in years; health system: health care type (subsidized, contributory); geographic: place of residence; comorbidities and health habits: smoking, obesity, asthma, cancer, HIV, immunodeficiency, diabetes, cardiovascular diseases, chronic respiratory disease, chronic liver disease; initial symptoms: date of onset of symptoms, fever, cough, breathing difficulty, general tiredness, date of laboratory confirmation; case type (recovered, death); place of care provision: outpatient clinic, hospital (hospitalization in a general ward, intensive care unit (ICU), hospitalization in intermediate care), and home care.

Direct medical costs were estimated by place of care provision, describing the costs of consultations, laboratory and imaging examinations, material, medication, procedures, medical transport, household care, hospitalization in a general ward, hospitalization in intermediate care and hospitalization in an ICU.

*Data relating to direct and indirect costs*

At the time of this study, there were no publications on out-of-pocket expenses or indirect costs of COVID-19 in the population of interest. For this reason, we used the mean out-of-pocket expenses and indirect costs [US$ 168 (SD 15.4)] estimated in 44 pediatric patients hospitalized for severe acute respiratory failure in Cartagena (near the Department of Córdoba) as a proxy for direct expenses and indirect costs of COVID-19.^8^ In this sense, considering a population of 4,267 COVID-19 cases confirmed by the EPS in Córdoba and an estimation error of US$3 or 1.17%, with a 5% significance level %, considering a normal probability distribution, the estimated sample size was 100 patients (Supplementary Figure 1). The sampling procedure and randomization were stratified by health care type and whether or not the cases were hospitalized. 

The direct and indirect costs associated with COVID-19 were obtained through a structured and standardized questionnaire, with interviews carried out by telephone in the first seven days after outpatient recovery (mild cases with home care) or hospital discharge (severe/critical cases). The questionnaire included questions about personal or family expenses. 

*Variables used to estimate non-medical direct costs (out-of-pocket expenses) and indirect costs*

The interview conducted with COVID-19 cases included the following variables: person interviewed (patient, caregiver); socioeconomic data: age in years; sex (male, female); health care type (subsidized, contributory); municipality of residence; household socioeconomic bracket (low-low, low, medium-low); schooling (complete high school education, higher education, complete elementary education, technician or technologist, incomplete high school education, incomplete primary education, none, postgraduate education); professional status and family income: occupation prior to the event (working, not working, student, domestic service, other); family income (less than 1 minimum wage, 1-2 minimum wages, 2-3 minimum wages), benefits, considering oneself to be poor; health characteristics (any comorbidity, smoker or former smoker); nutritional status (normal, overweight, obesity); COVID-19 care (outpatient, emergency, hospital), disease duration (days), and case type (symptomatic, asymptomatic). Out-of-pocket expenses and indirect costs were estimated by asking about expenditure on food, copayment, image laboratory, medication, supplements, transport, family income and disease duration.

*Statistical analysis*

Analysis of estimated direct medical costs from the perspective of the health system was carried out using records of health services billed according to patient and context of care. These were classified into cost aggregation groups using the bottom-up costing method.^9^ Formally, direct medical costs were estimated as follows:

$$\forall \text{i}\in \text{K}:\text{Patient cost items}=h_{i}\times p_{i}$$

Where:

$$\text{K}=\text{Medical visits, Examinations, Hospitalizations, Procedures},\ldots ,h_{i}$$

As such, direct medical cost is:

$$\text{Direct cost}=\sum_{j=1}^{k}h_{i}\times p_{i}$$

In turn, a generalized linear model (GLM) was estimated with gamma distribution and logarithmic link function to model the mean cost, seeking associated factors. These can be expressed as follows:

$$\begin{array}{c} E(y|x)=g^{-1}(x\beta )\\ where:y∼F\\ \partial E(y|x)/\partial x=\hat{\beta }[(\partial E(y|x)/\partial g)g^{-1}(x\hat{\beta }+\varepsilon )] \end{array}$$

Where the function g(.) is the link with the mean costs and the explanatory variables, F is the distribution of the exponential family, in this case the variable follows a gamma probability distribution and ∂y/∂x=β is the incremental cost. It was assumed that direct medical costs followed a gamma distribution, commonly used in cost function estimation studies^10^ ^)-(^ ^12^ in the presence of patients with extreme costs and long tails to the right of the distribution. Adjustment statistics, cost ratio estimates obtained by exp(β) and the mean marginal effect of the incremental cost increase are reported. All variables from the database were included in the model, without selection based on statistical criteria. Estimates were generated with robust errors using White’s estimator.^13^

To estimate direct non-medical costs, as with direct medical costs, the total expense for each component was quantified by multiplying the purchase price by the quantities purchased.

To estimate indirect costs, lost productivity or the opportunity cost approach due to illness care was considered. To this end, caregivers and/or patients were asked to estimate the time spent caring for the person with COVID-19. Productivity loss was estimated as income lost resulting from the number of days of paid work and the caregiver’s and/or patient’s salary. Indirect costs for students, housewives and the unemployed were estimated using the time spent caring for the patient multiplied by the 2020 Colombian legal daily minimum wage as an indicator of the price, in the form of opportunity cost, of the time spent in other activities, instead of being active on the job market.^8^

Estimates of economic costs were calculated by summing direct medical costs, direct non-medical costs and indirect costs. For each economic cost component, summary statistics were estimated based on the individual cost, such as means, standard deviation, the respective 95% confidence interval (95%CI), median and interquartile ranges (IQR). Descriptive statistics indicated the individual mean or median cost in each component and their aggregation estimated the mean or median economic cost of a representative episode of COVID-19 in the Department of Córdoba. Finally, the estimated economic cost for a COVID-19 event was extrapolated to all cases in the Department, through bootstrapping or resampling in a thousand iterations of a thousand possible economic cost scenarios assuming a normal probability distribution, thus allowing the total cost in the period to be estimated. All costs were expressed in dollars, using the representative market rate at the end of October 2020 (3,858.6 COP/US$).^14^

Orthogonal contrast tests and regression models were estimated at 5% significance. The analyses were performed in R version 4.0.3 (2020-10-10) using the following packages: *tidyverse*, *gtsummary*, *dplyr*, *ggplot2*, *R base (glm)*, *flextable.*

*Ethical considerations*

The study and its data collection instruments were reviewed and approved, so this work was categorized as “risk free”^15^ by the research ethics subcommittee of the Instituto de Investigaciones Biológicas del Trópico da Universidade de Córdoba, as per Opinion no. 0224-2021. Furthermore, the study followed the guidelines for research involving human subjects established in the Declaration of Helsinki^16^ and the International Ethical Guidelines for Health-Related Research Involving Human Subjects published by CIOMS.^17^

## RESULTS

*Estimated direct medical costs*

We studied 1,800 confirmed COVID-19 cases. Mean age was 45.2 (SD = 20.9) years, the majority were female (970; 53.9%) and subsidized health subscribers (1,415; 78.6%). Mean stay was 8.6 (SD = 7.5) days and 10.9 in ICUs (SD = 8.8) (Table 1).

**Table 1:** ‒ Characterization of recovered COVID-19 patients with health service demands in the Department of Cordoba, Colombia, 2020 and 2021

Variable	Total (n = 1,800)
Age in years, Mean (SD)	45.2 (20.9)
Sex	
Female	970 (53.9)
Male	830 (46.1)
Health care type	
Subsidized	1,415 (78.6)
Contributory	385 (21.4)
Notifying municipality	
Montería	851 (47.3)
Tuchín	175 (9.7)
Cereté	157 (8.7)
Lorica	114 (6.3)
Planeta Rica	84 (4.7)
Ciénaga de Oro	79 (4.4)
Other municipalities	340 (18.9)
Health history	
Smoker	6 (0.3)
Any comorbidity	218 (12.1)
Obesity	27 (1.5)
Asthma	14 (0.8)
Cancer	12 (0.7)
HIV	4 (0.2)
Immunodeficiency	1 (0.1)
Diabetes	124 (6.9)
Cardiovascular diseases	54 (3.0)
Chronic respiratory disease	8 (0.4)
Chronic liver disease	1 (0.1)
Initial symptoms	
Any symptom at onset	962 (53.4)
Cough	591 (32.8)
Fever > 38ºC	556 (30.9)
Breathing difficulty	400 (22.2)
General tiredness	519 (28.8)
Follow-up and result, mean (SD)^a^	
Observation time (days)	34.7 (23.1)
Case type	
Recovered	1,613 (89.6)
Death	187 (10.4)
Health care characteristics	
Place of care provision^b^	
Outpatient clinic	1,077 (59.8)
Hospital	903 (50.2)
Home care	441 (24.5)
Patients cared for by type of hospital bed	
Hospitalization in general ward	346 (75.9)
Hospitalization in intensive care unit (ICU)	75 (16.4)
Hospitalization in intermediate care	35 (7.7)
Mean length of inpatient stay (SD)	8.6 (7.5)
Mean hospital inpatient stay days General ward (SD)	7.6 (6.8)
Mean (SD) inpatient stay days in intermediate care (SD)	10 (6)
Mean inpatient stay days in intensive care unit (ICU) (SD)	10.9 (8.8)

a) Per 100,000 inhabitants (age groua a) SD: Standard deviation; b) Patients may have sought services in more than one care setting, the sum of ill people exceeds the total. p of 10-19 years).

Table 2 presents the mean and median cost per group of services. The mean direct medical cost of a COVID-19 event was estimated at US$ 2,342 (95%CI 1,892;2,778). Of this cost, 19.9% related to outpatient medical services, 6.4% to household care and 73.7% to hospital costs.

**Table 2:** ‒ Mean cost and 95% confidence interval (95%CI) cost of a COVID-19 event in the Department of Córdoba, Colombia, between 2020 and 2021

Type of cost	N	Mean cost ($)	Lower 95%CI ($)	Upper 95%CI ($)	Median cost ($)	25% IQR^a^ ($)	75% IQR^a^ ($)
Direct costs	1,800	2,391.5	1,925.4	2,844.4	1,299.4	1,120.4	2,310.9
Direct medical costs	1,800	2,342	1,892	2,778	1,274	1,107	2,246
Outpatient	1,077	463.8	205.7	708.7	334.9	259.3	487.1
Consultations	55	29.7	14.5	44.9	11.5	4.2	21.0
Laboratory and imaging examinations	1,035	20.7	19.1	22.2	10.1	10.1	20.1
Material	5	51.9	36.1	67.7	42.8	42.8	47.3
Medication	1	6.6			6.6	6.6	6.6
Procedures	8	233.7	64.6	402.9	180.2	125.1	231.2
Surgical procedures	5	56.0	27.7	84.2	50.7	50.7	50.7
Medical transport	25	65.2	43.7	86.8	33.0	19.8	110.2
Household	903	150.3	146.2	154.5	175.7	85.3	202.7
Household care	903	150.3	146.2	154.5	175.7	85.3	202.7
Hospitalization	441	1,727	1,540	1,915	762.9	762.9	1,556
Hospitalization in general ward	346	1,078	942.8	1,213	762.9	762.9	956.1
Hospitalization in intermediate care	35	2,374	1,655	3,094	1,332	1,332	2,749
Hospitalization in ICU	75	4,076	3,520	4,632	4,338	3,090	4,338
Direct expenses	57	49.9	33.6	66.2	25.9	13.0	64.8
Food	3	8.6	4.2	13.1	7.8	6.5	10.4
Copayment	2	2.0			2.0	1.4	2.5
Image laboratory	4	45.4	13.7	77.0	38.9	23.3	60.9
Other medical	19	30.4	15.7	45.2	14.8	11.7	37.6
Medication	46	32.6	20.0	45.2	19.4	8.1	38.9
Supplements	7	35.5	17.0	54.1	20.7	16.8	51.8
Transport	20	9.5			1.9	1.2	4.2
Indirect costs	66	127.8	53.1	202.5	47.9	20.0	123.2
Indirect family costs	22	152.8	3.9	301.7	22.0	7.8	129.6
Indirect patient costs	56	90.6	54.6	126.6	47.9	22.3	110.2
Economic cost	1,899	2,519	1,979	3,047	1,347	1,140	2,434

a) Interquartile range.

Note: The blank 95%CI cells are not significant.

**Figure 1:**
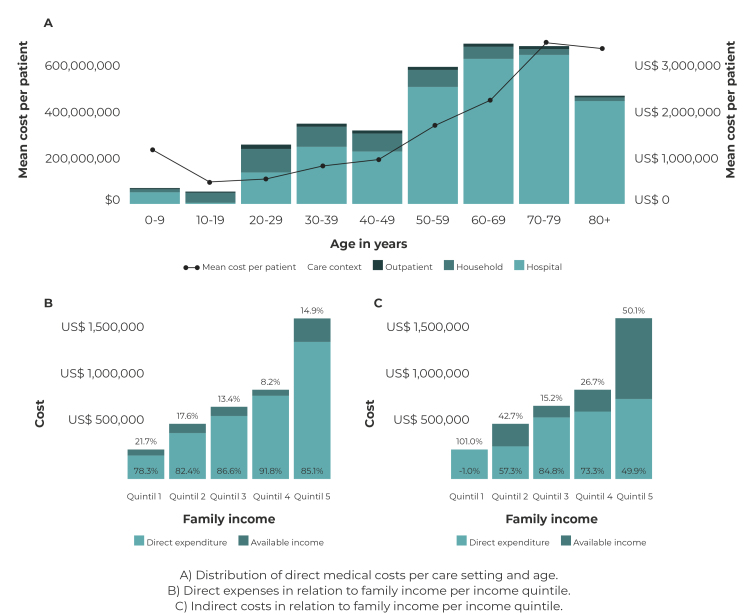
‒ Direct medical costs per care setting and age, direct expenses and indirect costs in relation to family income per income quintile attributable to COVID-19 no Department of Córdoba-Colombia, between 2020 and 2021

Note: The information relating to direct medical costs are for the period March-September 2020. The information relating to out-of-pocket expenses and indirect costs are for the period January-February 2021.

In most age groups, hospitalized patients accounted for the majority of total medical costs, increasing proportionally with age. Cumulative total costs were concentrated in 53% of individuals aged 50 or over, a group that accounts for 27% of cases (Figure 1A).

The generalized linear model (Table 3) identified that factors such as age (additional 2% increase in cost per year), having a comorbidity (2.1 times) and being symptomatic (3.6 times) significantly increase the mean direct medical cost of COVID-19 compared to those without these characteristics. These factors remained significant for hospital costs. On the other hand, the subsidized subscription type and the cases reported in Montería resulted in an increase in total outpatient and home care costs, but not in hospital costs.

**Table 3:** ‒ Model of direct medical cost ratios attributed to COVID-19 and 95% confidence intervals (95%CI) in affected people in Córdoba, Colombia, 2020 and 2021

Variables	Cost ratio^a^	Lower 95%CI	Upper 95%CI	p-value	Marginal effects at the means	Lower 95%CI	Upper 95%CI	p-value
Interception	23.56	17.45	31.81	< 0.001				
Sex: Male	1.05	0.87	1.28	0.601	26.96	-79.65	133.58	0.620
Age (years)	1.02	1.01	1.03	< 0.001	9.82	6.50	13.14	< 0.001
Health care type Subsidized	2.11	1.55	2.87	< 0.001	300.21	208.54	391.87	< 0.001
Case from the capital	1.62	1.32	1.99	< 0.001	245.42	135.14	355.70	< 0.001
Comorbidity	2.05	1.57	2.69	< 0.001	457.88	191.13	724.64	0.001
Symptomatic	3.56	2.87	4.42	< 0.001	456.77	369.90	543.65	< 0.001
N	1.800							
Nagelkerke’s pseudo R^2^	0.39							
Log likelihood	-12 165.76							

a) Robust errors.

Regarding marginal effects, an additional year of a patient with COVID-19 increases the cost of care by US$ 9.8 (95%CI 6.5;13.1). Furthermore, the presence of at least one comorbidity implied an additional mean cost of US$ 457.9 (95%CI 191.1;724.6) compared to those without comorbidities (Table 3).

*Estimate of direct non-medical costs and indirect costs*

Table 4 shows the sociodemographic characteristics of the 99 COVID-19 cases interviewed. Of these, 57.6% reported out-of-pocket expenses related to COVID-19 care. The mean amount of direct expenses borne by patients and families was US$ 49.9 (95%CI 33.6;66.2); of these expenses, 55% were on medication (Table 2).

**Table 4:** ‒ Characterization of recovered COVID-19 cases, interviewed regarding direct expenses and loss of productivity, Córdoba, Colombia, between 2020 and 2021

Variable	Total (n = 99)
Person who took part in the interview	
Patient	68 (68.7)
Caregiver	31 (31.3)
Socioeconomic data	
Age. years. mean (SD)	42.9 (21.7)
Sex	
Female	56 (56.6)
Male	43 (43.4)
Health care type	
Subsidized	83 (83.8)
Contributory	16 (16.2)
Municipality of residence	
Montería	47 (47.5)
Lorica	16 (16.2)
Planeta Rica	5 (5.1)
Cereté	4 (4.0)
Chinú	3 (3.0)
Cotorra	3 (3.0)
Sahagún	3 (3.0)
Other municipalities	18 (18.2)
Household socioeconomic bracket	
Low-low (1)	76 (76.8)
Low (2)	22 (22.2)
Medium-low (3)	1 (1.0)
Schooling	
Complete high school education	25 (25.3)
Higher education	17 (17.2)
Complete elementary education	13 (13.1)
Technician or technologist	13 (13.1)
Incomplete high school education	12 (12.1)
Incomplete primary education	11 (11.1)
None	7 (7.1)
Postgraduate education	1 (1.0)
Professional status and family income	
Occupation before the COVID-19 event	
Working	55 (55.6)
Not working	16 (16.2)
Student	14 (14.1)
Domestic service	10 (10.1)
Other	4 (4.0)
Currently working	
No	69 (69.7)
Yes	30 (30.3)
Reason for being unemployed	
Other reason	10 (41.7)
Voluntary change due to the disease	7 (29.2)
Termination, contract not renewed	6 (25.0)
Family members’ request	1 (4.2)
Number of people in the family, mean (SD)	4.2 (1.7)
Family income	
Less than 1 minimum wage	75 (75.8)
1-2 minimum wages	22 (22.2)
2-3 minimum wages	2 (2.0)
Did any family member receive benefits	
No	65 (65.7)
Yes	34 (34.3)
Does family income cover expenses	
No	89 (89.9)
Yes	10 (10.1)
Do you consider yourself to be poor	
Yes	66 (66.7)
No	33 (33.3)
Health characteristics	
Any comorbidity	46 (46.5)
Smoker or former smoker	17 (17.2)
Nutritional status	
Normal	80 (80.8)
Overweight	15 (15.2)
Obesity	4 (4.0)
COVID-19 care^a^	
Outpatient	90 (90.9)
Emergency	10 (10.1)
Hospital	8 (8.1)
Disease duration (days), mean (SD)	11.1 (13.4)
Case type	
Symptomatic	53 (53.5)
Asymptomatic	46 (46.5)

a) People may have sought services in more than one care setting, the sum of ill people exceeds the total.

On the other hand, 66.7% of respondents mentioned indirect costs. Mean time spent on COVID-19 was 11.1 days (SD = 13.4) and the mean time spent by family members was 15.4 days (SD = 21.6). Of the patients, 86.9% were of legal age and 57.6% had a paid activity with an estimated mean monthly income of US$ 233.1 (SD = 125.6). The indirect cost due to COVID-19 was estimated at US$ 127.8 (95%CI 53.1;202.5) (Table 2).

Mean family size was 4.2 people (SD = 1.7), with a mean monthly income of US$ 204.4 (SD = 121.3), representing a mean of 16.5% of family income. In terms of income quintiles, this proportion increased for family members with lower incomes, reaching 21.7% (Figure 1B). Indirect costs reflect the estimated loss of family income due to the disease, representing 54.2% of income; a proportion that, in the lowest quintile, represented 101% of total family income (Figure 1C).

Family members turned to different sources of financing to cover expenses incurred due to COVID-19. Of these family members, 39.4% used savings, 35.4% other sources, 27.3% borrowed money and 10.1% needed donations. In the lowest income quintile, use of savings was the largest source of financing, accounting for 45%.

*Estimated economic cost*

The estimated mean economic cost of COVID-19 in the Department of Córdoba was US$ 2,519 (95%CI 1,980;3,047), 92.9% comprised of direct medical costs. In turn, median economic cost was US$ 1,347 (IQR 1,140;2,434) (Table 2). On April 14, 2020, the National Institute of Health reported a total of 46,233 confirmed cases of COVID-19 in the Department of Córdoba,^2^ so the economic cost extrapolated to total cases would be between US$ 116.5 and US$ 118.1 million.

## DISCUSSION

This study estimated a mean economic cost of COVID-19 per episode in the Department of Córdoba of US$ 2,519 (95%CI 1,979;3,047), 92.9% for direct medical costs; direct and indirect expenses accounted for 2% and 5.1%, respectively. Based on this cost, we estimated that the economic burden for the Department represented between 2.4% and 2.5% of its 2019 Gross Domestic Product (GDP) (US$ 4,741 million).^18^ In *per capita* terms (US$ 2,622), the economic cost of an average COVID-19 event in Córdoba would account for between 75.5% and 116.2%.

The direct expenses estimated in this study represent, on average, 16.5% of family income, with a greater burden in the lowest income quintile (21.7%). In turn, indirect costs reflect the estimated loss of family income resulting from the disease, representing 54.2% of income. According to the Monetary Deficiency Measure (2019), Córdoba was one of three Departments with the lowest poverty bracket in Colombia (US$ 67.8) and the second lowest extreme poverty bracket (US$ 31.5), evidenced by the percentage of the population with an expenditure unit *per capita* income below these limits (54.2% and 15.2%, respectively).^19^ The direct expenses estimated in this study indicate that 277,000 people in the Department would not be able to cover the cost of the disease with the income they had (taking the 2020 population). In terms of lost productivity, 991,000 people in the Department would potentially see their income and that of their family members being affected by COVID-19.

Loss of income and the burden on the most vulnerable in the face of COVID-19 presents a greater dimension if we consider the loss of jobs due to restrictions in all economic sectors. During 2020, Córdoba had an unemployment rate of 15.7%, while Montería had an informal labor rate of 59.9%.^20^ Regarding this, Bonnet et al.^21^ and Ricciulli-Marín et al.^22^ estimated the regional impact of COVID-19, showing how mobility restrictions in Córdoba would imply losses of 5.7% of GDP, mainly in the civil construction and other services sectors. In fact, in this study, 49.1% of people who said they worked before contracting COVID-19 declared that they were not working at the time of the interview. Of the latter, 25% indicated that the cause was dismissal or non-renewal of their employment contract and a further 29.2% stated that they no longer worked due to the disease.

Regarding the mean direct medical cost, this is largely explained by hospitalization, in which cases with hospital admission had costs 16.2 times higher than less serious ones. Our estimate was similar to the US$ 2,283 (IQR 788;2,523) calculated by Alvis-Zakzuk et al. for an average case in Colombia.^23^ Other estimates, such as that made by Bartsch et al.,^24^ indicated a mean direct medical cost of US$ 3.045. In turn, Li et al.,^25^ discovered through micro-costing that a patient cost a mean of US$ 6,827 per event, with the cost concentrated on medication (45% of the mean cost). Regarding hospital costs, our results indicate a mean hospitalization cost in a general ward of US$ 1,078 (95%CI 942.8;1,213) and a mean ICU cost of US$ 4,076 (95%CI 3,520;4,632). Similar results were published by Khan et al.,^26^ who found a mean hospitalization cost per patient of US$ 1.385 (SD = 166.04).

Mean direct medical cost varies depending on factors such as age, comorbidities and symptoms. We found that health care type and case notification in the capital appeared to increase total cost and outpatient and home costs, but not hospital costs. This result shows the conditions of access to household care and outpatient services in the capital compared to other municipalities, together with a concentration of cases in the capital (47.3% in Montería). Since direct medical expenses were collected from an insurance company in which subscribers on the subsidized health care type are the majority, the results reflect the sample imbalance between the two subscription types and the uniqueness of the insurance company.

This study has important limitations to be considered. Firstly, the estimated results are partial, given the study period. Secondly, some records on the available databases were incomplete, which could underestimate medication-related costs in the direct medical cost component. This limitation could be mitigated by expanding the sample of COVID-19 cases covered by other insurance companies, reducing estimation bias and increasing the accuracy of results. A third limitation is the fact that the estimated economic costs did not take into account the costs associated with lost working hours or layoffs related to the measures taken by the population to limit the transmission of the virus during the pandemic (mandatory preventive isolation) and the time spent by caregivers or family members who cared for the ill. In fact, these population measures generated economic losses estimated between US$ 1.2 million and US$ 15.3 million per month of isolation, equivalent to 0.5% and 6.1% of national GDP.^21^ It should be noted that this study does not present costs of long COVID-19. It is important, in future analyses, to estimate the subsequent costs due to impairments caused by COVID-19, in order to complete the estimate of the economic burden of this disease. Another limitation is potential recall bias when reporting costs in the past via telephone interviews. Asking for past expenses could result in underestimated out-of-pocket expenses. However, a period of seven days is not so long as to be significant.^27^

Given the significant economic impact of COVID-19, especially on low-income families and increasing job losses, health policies must focus on two crucial areas. First, specific attention needs to be paid to the financial protection of vulnerable families through financial support programs. Second, measures to preserve employment and income need to be implemented, such as employment support and commercial subsidies. The interconnection between physical and economic health highlights the importance of a comprehensive approach to public health, addressing not only the prevention and treatment of disease, but also its social and economic impacts, with measures to mitigate inequalities. 

Estimating the costs of an illness is a necessity for any healthcare system, especially those related to out-of-pocket expenses and indirect costs related to lost productivity. These are the main inputs for evaluating the cost-effectiveness of new health interventions to help mitigate the effects of the disease. In this sense, the methods used in this article can be replicated in other health systems and in other latitudes. Future analyses can assess the costs of illness for COVID-19, long COVID and related respiratory illnesses, taking into account the nature of the healthcare system (private and public), geographic variability and socioeconomic inequalities in the population. 

This study demonstrated the high economic burden faced by the health system and families in the Department of Córdoba due to COVID-19. The most vulnerable families had to bear a large part of the burden on their income, with important consequences for social progress and overcoming extreme poverty.
