# Supplementary material for: Economic burden due to COVID-19 in a Colombian Caribbean state, 2020 and 2021
Source: Epidemiol Serv Saude. 2024 Jul 8;33:e2023830. doi: 10.1590/S2237-96222024V33E2023830.EN (PMC11251452; doi:10.1590/S2237-96222024V33E2023830.EN)
Supplement: Translation

# Translation: Carga econômica associada à covid-19 em um departamento do Caribe colombiano, 2020 e 2021

**DOI:** 10.1590/S2237-96222024V33E2023830.PT

**Authors:** Fernando Salcedo-Mejía, Lina Moyano-Tamara, Josefina Zakzuk, Ana Milena Lozano, Héctor Serrano-Coll, Bertha Gastelbondo, Salim Mattar Velilla, Nelson Rafael Alvis Zakzuk, Nelson J. Alvis-Zakzuk, Nelson Alvis Guzmán

**Affiliations:** 1 ALZAK Foundation, Cartagena, Bolívar, Colômbia; 2 Universidad de Cartagena, Grupo de Investigación en Economía de la Salud, Cartagena, Bolívar, Colômbia; 3 Universidad CES, Instituto colombiano de Medicina tropical, Apartadó, Colômbia; 4 Universidad de Córdoba, Instituto de Investigaciones Biológicas del Trópico, Montería, Córdoba, Colômbia; 5 Universidad de la Costa, Departamento de Ciencias de la Salud, Barranquilla, Atlántico Colômbia

**Keywords:** Custos de Cuidados de Saúde, Custo da Doença, Carga Econômica, Covid-19

## Abstract

**Objetivo:**

Estimar a carga econômica associada à covid-19 em Córdoba, Colômbia, entre 2020 e 2021.

**Métodos:**

Estudo de avaliação da carga econômica da covid-19. Os custos diretos foram analisados segundo terceiros pagadores, usando-se bases de dados administrativas e entrevistas. A agregação dos custos foi realizada usando-se o método *bottom-up*. Os custos indiretos foram estimados pela perda de produtividade. Testes de contraste ortogonais e modelos de regressão estatísticos foram estimados com 5% de significância.

**Resultados:**

Foram estudadas 1.800 pessoas com covid-19. O custo econômico médio da covid-19 por episódio foi estimado em US$ 2.519 (IC_95%_ 1.980;3.047). O componente de custo médico direto representou 92,9% do total; as despesas diretas e custos indiretos representaram 2,0% e 5,1%, respectivamente.

**Conclusão:**

O custo econômico da covid-19 deveu-se principalmente aos custos médicos diretos. Este estudo forneceu avaliações da carga econômica enfrentada pelas famílias devido à covid-19; as mais vulneráveis tiveram grande parte da carga sobre os seus rendimentos.

## Contribuições do estudo

**Principais resultados:**

O custo econômico médio da covid-19 por episódio foi estimado em US$ 2.519 (IC_95%_ 1.980;3.047). O componente de custo médico direto representou 92,9% do total; as despesas diretas e custos indiretos, 2% e 5,1%, respectivamente.

**Implicações para os serviços:**

Estimar os custos de uma doença é necessário para qualquer sistema de saúde, especialmente as despesas próprias e custos indiretos. Esses são os principais insumos para avaliar o custo-efetividade de novas intervenções de saúde.

**Perspectivas:**

É importante, em análises futuras, estimar os custos subsequentes causados pela incapacidade gerada pela covid-19 longa, a fim de se completar a estimativa da carga econômica dessa doença.

## INTRODUÇÃO

Entre dezembro de 2019, quando a covid-19 foi identificada, e meados de abril de 2021, ocorreram mais de 411 milhões de casos em todo o mundo e aproximadamente 2,9 milhões de mortes.^1^ Na Colômbia, até 14 de abril de 2021, foram notificados 2.585.801 casos e 66.819 mortes por covid-19, e, enquanto algumas cidades já estavam vivenciando seu terceiro pico, outras estavam no segundo.^2^ Foram registradas também diferenças regionais nas taxas de incidência, mortalidade e letalidade.^2^ Esse fato, associado às especificidades de cada região, torna cada departamento único, no que diz respeito ao impacto da pandemia.

Os efeitos da covid-19 sobre os sistemas de saúde repercutiram na capacidade de cobrir a pandemia e, ao mesmo tempo, de manter a oferta desses serviços.^3^ Tais efeitos seriam ainda maiores em países de baixa e média renda, com custos significativos na saúde. Foi estimado que os custos da resposta à covid-19, em um cenário de quatro semanas, foram de aproximadamente US$ 52 bilhões, a uma taxa de US$ 8,60 *per capita*.^4^ Por sua vez, as famílias enfrentaram não só perdas inesperadas de emprego e renda durante a pandemia, mas também despesas médicas para cuidar dos membros que contraíram a doença. Essas despesas diretas afetaram seus rendimentos já reduzidos, em todo o mundo, no contexto da pandemia.^5^ ^),(^ ^6^

A estimativa da carga econômica de uma doença permite fornecer informações sobre os custos médicos diretos, custos não médicos e custos indiretos, associados à sua gestão. Uma vez que as abordagens de análise de custos para a covid-19 na Colômbia ainda são escassas, é válido avaliar economicamente a frequência de utilização dos serviços de saúde e, especialmente, conhecer as despesas diretas e os custos associados à perda de produtividade, que, normalmente, não são estimados em estudos de custo de uma enfermidade. Por essa razão, o objetivo deste trabalho foi estimar a carga econômica associada à covid-19 em Córdoba, Colômbia, em 2020 e 2021.

## MÉTODOS

*Desenho do estudo*

Trata-se de estudo de avaliação da carga econômica da covid-19, sob a perspectiva da sociedade, no período entre março de 2020 e abril de 2021.

*Local do estudo e contexto*

Córdoba é um departamento situado ao norte da Colômbia com elevada mortalidade por covid-19. Era constituído por 30 municípios e 1.838.574 habitantes em 2020.^7^ A capital, Montería, foi uma das cidades mais afetadas no início da pandemia. Em 14 de abril de 2021, registrava uma das incidências acumuladas mais baixas entre as principais cidades do Caribe colombiano, mas a taxa de mortalidade era a mais elevada da região.^2^

*Participantes*

Casos diagnosticados com covid-19 e tratados pelo sistema de saúde em Córdoba, Colômbia. A Figura Suplementar 1 apresenta o fluxograma da seleção dos casos de covid-19 para as estimativas de custos econômicos. Do total de casos de covid-19 em Córdoba, analisaram-se aqueles com frequências de uso de serviços de saúde para os custos diretos médicos, e uma subamostra foi selecionada para estimar as despesas próprias bolso e custos indiretos. 

*Fonte de dados e variáveis*

*Dados de custos médicos diretos*

Foram analisadas as bases de dados administrativas dos serviços de saúde de uma coorte de casos positivos de covid-19 em Córdoba. A Empresa Promotora de Salud (EPS), que gerou essas bases, identificou 4.267 casos de covid-19 no departamento até o final de 2020 (Figura Suplementar 1). Os seus afiliados, que pertencem a ambos os regimes do sistema de saúde colombiano [contributivo (regime que afilia os trabalhadores ou a população com capacidade de pagamento) e subsidiado (regime que afilia pessoas com baixa renda)], correspondiam a 30,2% da população do departamento em 2020. Então foram recolhidas informações sobre a sintomatologia, gravidade e comorbilidades, serviços e custos em ambulatório, internação domiciliar e hospitalização em clínicas e hospitais do departamento.

*Variáveis para custos diretos médicos*

As variáveis disponíveis foram: demográficas: sexo (feminino, masculino), idade em anos; sistema de saúde: regime de saúde (subsidiado, contributivo); geográficas: local de residência; comorbidades e hábitos de saúde: fumante, obesidade, asma, câncer, HIV, imunodeficiência, diabetes, doenças cardiovasculares, doença respiratória crônica, doença hepática crônica; sintomas iniciais: data de início dos sintomas, febre, tosse, dificuldade respiratória, fadiga geral, data de confirmação laboratorial; tipo de caso (recuperado, óbito); local de prestação de cuidados: ambulatorial, hospitalar (hospitalização geral, unidade de terapia intensiva UTI, hospitalização em cuidados intermediários), e assistência domiciliar.

Os custos diretos médicos foram estimados por local de prestação de cuidados, descrevendo- -se os custos de consultas, laboratório e exames de imagem, materiais, medicamentos, procedimentos, transporte médico, cuidados domiciliares, hospitalização geral, internação em cuidados intermediários e hospitalização em UTI.

*Dados relativos aos custos diretos e indiretos*

Na época deste estudo, não havia publicações sobre as despesas próprias ou custos indiretos com a covid-19 na população de interesse. Por esse motivo, usamos a média das despesas próprias e custos indiretos [US$ 168 (DP 15,4)] estimados em 44 pacientes pediátricos hospitalizados por insuficiência respiratória aguda grave (SARI) em Cartagena (perto do departamento de Córdoba) como uma *proxy* para as despesas diretas e custos indiretos da covid-19.^8^ Nesse sentido, considerando-se uma população de 4.267 casos confirmados de covid-19 na EPS em Córdoba e um erro de estimativa de US$ 3 ou 1,17%, com um nível de significância de 5%, considerando-se uma distribuição de probabilidade normal, a amostra estimada foi de 100 pacientes (Figura Suplementar 1). O procedimento amostral e a aleatorização foram estratificados por regime de saúde e pelo fato de os casos estarem ou não hospitalizados. 

Os custos diretos e indiretos associados à covid-19 foram obtidos através de um questionário, estruturado e normatizado, com as entrevistas realizadas por telefone nos primeiros sete dias após a recuperação ambulatorial (casos leves com cuidados de internação domiciliar) ou a alta hospitalar (casos graves/críticos). O questionário incluiu questões sobre despesas pessoais ou familiares. 

*Variáveis usadas na estimativa dos custos diretos não médicos (despesas próprias) e custos indiretos*

A entrevista feita com as pessoas com covid-19 incluiu as variáveis que seguem: pessoa entrevistada (paciente, cuidador); dados socioeconômicos: idade em anos; sexo (masculino, feminino); regime de saúde (subsidiado, contributivo); município de residência; faixa socioeconômica do domicílio (baixo-baixo, baixo, médio-baixo); escolaridade (ensino médio completo, superior, ensino fundamental completo, técnico ou tecnológico, ensino médio incompleto, primário incompleto, nenhum, pós-graduação); situação profissional e rendimento familiar: ocupação antes do evento (trabalhava, não trabalhava, estudava, trabalho doméstico, outra); rendimento familiar (menos de 1 salário mínimo, 1-2 salários mínimos, 2-3 salários mínimos), subsídios, considera-se pobre; características em saúde (alguma comorbilidade, fumante ou ex-fumante); estado nutricional (normal, sobrepeso, obesidade); atenção da covid-19 (ambulatório, emergência, hospitalar), tempo da doença (dias), e tipo de caso (sintomático, assintomático). As despesas próprias e os custos indiretos foram estimados perguntando-se pelas despesas em alimentação, copagamento, laboratório de imagem, medicamentos, suplementos, transporte, rendimento do familiar e tempo da doença.

*Análise estatística*

A análise dos custos médicos diretos estimados da perspectiva do sistema de saúde foi feita a partir do uso de registros de serviços de saúde faturados por paciente e pelo contexto de cuidados. Estes foram classificados em grupos de agregação de custos, utilizando-se o método de cálculo de custos ascendente (*bottom-up*).^9^ Formalmente os custos médicos diretos foram estimados da seguinte forma:

$$\forall \text{i}\in \text{K}:\text{Items custos paciente}=h_{i}\times p_{i}$$

Onde:

$$\text{K}=\text{Visitas médicas, Exames, Internações, Procedimentos},\ldots ,h_{i}$$

Assim, o custo direto médico é:

$$\text{Custo direto}=\sum_{j=1}^{k}h_{i}\times p_{i}$$

Por sua vez, foi estimado um modelo linear generalizado (MLG) com distribuição gama e função de ligação logarítmica para modelar o custo médio, buscando fatores associados. Estes podem ser expressos da seguinte forma:

$$\begin{array}{c} E(y|x)=g^{-1}(x\beta )\\ onde:y∼F\\ \partial E(y|x)/\partial x=\hat{\beta }[(\partial E(y|x)/\partial g)g^{-1}(x\hat{\beta }+\varepsilon )] \end{array}$$

Onde a função g(.) é a ligação com a média dos custos e as variáveis explicativas, F é a distribuição da família exponencial, neste caso a variável segue distribuição de probabilidade gama e ∂y/∂x=β, o custo incremental. Assumiu-se que os custos diretos médicos seguiam uma distribuição gama, habitualmente utilizada em estudos de estimação de funções de custo^10^ ^)-(^ ^12^ na presença de pacientes com custos extremos e longas caudas à direita da distribuição. São reportadas as estatísticas de ajuste, as estimativas de razão de custos obtida pelo exp(β) e o efeito marginal médio do aumento do custo incremental. Todas as variáveis do banco de dados foram incluídas no modelo, sem seleção com base em critérios estatísticos. As estimativas foram geradas com erros robustos, usando-se o estimador de White.^13^

Para estimar os custos diretos não médicos, como acontece com os custos médicos diretos, a despesa total com cada componente foi quantificada multiplicando-se o preço de compra pelas quantidades adquiridas.

Para estimar os custos indiretos, foi considerada a perda de produtividade ou a abordagem do custo de oportunidade devido aos cuidados com a doença. Para efeito, solicitou-se aos cuidadores e/ou aos pacientes que estimassem o tempo gasto para cuidar da pessoa com covid-19. A perda de produtividade foi estimada como a renda perdida resultante do número de dias de trabalho remunerado e do salário do cuidador e/ou do paciente. Os custos indiretos para os estudantes, as donas de casa e os desempregados foram estimados utilizando-se o tempo gasto para cuidar do paciente multiplicado pelo salário mínimo diário legal colombiano de 2020 como um indicador do preço, sob a forma de custo de oportunidade, do tempo gasto em outras atividades, em vez de a pessoa estar atuante no mercado de trabalho.^8^

As estimativas de custos econômicos foram calculadas pela soma dos custos diretos médicos, custos diretos não médicos e custos indiretos. Para cada componente de custo econômico, foram estimadas estatísticas sumárias a partir do custo individual, como médias, desvio-padrão, o respectivo intervalo de confiança de 95% (IC_95%_), mediana e intervalos interquartis. A estatística descritiva indicou o custo médio ou mediano individual em cada componente e a agregação deles estimou o custo econômico médio ou mediano de um episódio representativo de covid-19 no departamento de Córdoba. Finalmente, o custo econômico estimado para um evento de covid-19 foi extrapolado para todos os casos no departamento, por meio de *bootstrapping* ou reamostragem em mil iterações de mil possíveis cenários de custo econômico, assumindo-se uma distribuição de probabilidade normal, o que permitiu estimar o custo total no período. Todos os custos foram expressos em dólares americanos (US$), utilizando-se a taxa de mercado representativa do final de outubro de 2020 (3.858,6 COP/US$).^14^

Os testes de contraste ortogonais e os modelos de regressão foram estimados com 5% de significância. As análises foram efetuadas em R versão 4.0.3 (2020-10-10), usando-se os pacotes *tidyverse*, *gtsummary*, *dplyr*, *ggplot2*, *R base (glm)*, *flextable.*

*Considerações éticas*

O estudo e seus instrumentos de coleta de dados foram revisados e aprovados, pelo que este trabalho foi categorizado, “sem risco”,^15^ pelo subcomitê de ética em investigação do Instituto de Investigaciones Biológicas del Trópico da Universidade de Córdoba, na Ata n°0224-2021. Além disso, o estudo seguiu as diretrizes de investigação envolvendo seres humanos estabelecidas na Declaração de Helsinque^16^ e os *International Ethical Guidelines for Health-Related Research Involving Human Subjects,* publicados pelo CIOMS.^17^

## RESULTADOS

*Estimativa dos custos médicos diretos*

Foram estudadas 1.800 pessoas confirmadas para a covid-19. A média de idade foi de 45,2 (DP = 20,9) anos, a maioria era do sexo feminino (970; 53,9%) e do regime subsidiado (1.415; 78,6%). A média de permanência hospitalar foi de 8,6 (DP = 7,5) dias e de 10,9 em UTI (DP = 8,8) (Tabela 1).

**Tabela 1:** ‒ Caracterização dos pacientes com covid-19 recuperados com demandas de serviços de saúde no departamento de Córdoba, Colômbia, 2020 e 2021

Variável	Total (n = 1.800)
Idade em anos, Média (DP)	45,2 (20,9)
Sexo	
Feminino	970 (53,9)
Masculino	830 (46,1)
Regime de saúde	
Subsidiado	1.415 (78,6)
Contributivo	385 (21,4)
Município de notificação	
Montería	851 (47,3)
Tuchín	175 (9,7)
Cereté	157 (8,7)
Lorica	114 (6,3)
Planeta Rica	84 (4,7)
Ciénaga de Oro	79 (4,4)
Outros municípios	340 (18,9)
Antecedentes	
Fumante	6 (0,3)
Qualquer comorbidade	218 (12,1)
Obesidade	27 (1,5)
Asma	14 (0,8)
Câncer	12 (0,7)
HIV	4 (0,2)
Imunodeficiência	1 (0,1)
Diabetes	124 (6,9)
Doenças cardiovasculares	54 (3,0)
Doença respiratória crônica	8 (0,4)
Doença hepática crônica	1 (0,1)
Sintomas iniciais	
Qualquer sintoma no início	962 (53,4)
Tosse	591 (32,8)
Febre > 38ºC	556 (30,9)
Dificuldade respiratória	400 (22,2)
Fadiga geral	519 (28,8)
Acompanhamento e resultado, média (DP)^a^	
Tempo de observação (dias)	34,7 (23,1)
Tipo de caso	
Recuperado	1.613 (89,6)
Óbito	187 (10,4)
Características dos cuidados de saúde	
Local de prestação de cuidados^b^	
Ambulatorial	1.077 (59,8)
Hospitalar	903 (50,2)
Assistência domiciliar	441 (24,5)
Pacientes atendidos por tipo de leito	
Hospitalização geral	346 (75,9)
Hospitalização em unidade de terapia intensiva (UTI)	75 (16,4)
Hospitalização em cuidados intermediários	35 (7,7)
Duração média da internação (DP)	8,6 (7,5)
Média de dias de permanência no hospital em internação geral (DP)	7,6 (6,8)
Média (DP) de dias de internação em cuidados intermediários (DP)	10 (6)
Dias de internação na UTI média (DP)	10,9 (8,8)

a) DP: Desvio-padrão; b) Os pacientes podem ter procurado serviços em mais de um contexto de cuidado, a soma dos doentes é superior ao total.

Na Tabela 2 apresenta-se o custo médio e mediano por grupo de serviços. O custo médico direto médio de um evento de covid-19 foi estimado em US$ 2.342 (IC_95%_ 1.892;2.778). Desse custo, 19,9% foi em serviços médicos ambulatoriais, 6,4% em cuidados domiciliares e 73,7% em custos hospitalares.

**Tabela 2:** ‒ Custo médio e intervalo de confiança de 95% (IC_95%_) de um evento de covid-19 no departamento de Córdoba, Colômbia, 2020 e 2021

Tipo de custo	N	Custo médio ($)	IC_95%_ inferior ($)	IC_95%_ superior ($)	Custo mediano ($)	IIQ^a^ 25% ($)	IIQ^a^ 75% ($)
Custos diretos	1.800	2.391,5	1.925,4	2.844,4	1.299,4	1.120,4	2.310,9
Custos diretos médicos	1.800	2.342	1.892	2.778	1.274	1.107	2.246
Ambulatório	1.077	463,8	205,7	708,7	334,9	259,3	487,1
Consultas	55	29,7	14,5	44,9	11,5	4,2	21,0
Laboratório e exames de imagem	1.035	20,7	19,1	22,2	10,1	10,1	20,1
Materiais	5	51,9	36,1	67,7	42,8	42,8	47,3
Medicamentos	1	6,6			6,6	6,6	6,6
Procedimentos	8	233,7	64,6	402,9	180,2	125,1	231,2
Procedimentos cirúrgicos	5	56,0	27,7	84,2	50,7	50,7	50,7
Transporte médico	25	65,2	43,7	86,8	33,0	19,8	110,2
Domiciliar	903	150,3	146,2	154,5	175,7	85,3	202,7
Cuidados domiciliares	903	150,3	146,2	154,5	175,7	85,3	202,7
Hospitalização	441	1.727	1.540	1.915	762,9	762,9	1.556
Hospitalização geral	346	1.078	942,8	1.213	762,9	762,9	956,1
Internação em cuidados intermediários	35	2.374	1.655	3.094	1.332	1.332	2.749
Hospitalização em UTI	75	4.076	3.520	4.632	4.338	3.090	4.338
Despesas diretas	57	49,9	33,6	66,2	25,9	13,0	64,8
Alimentação	3	8,6	4,2	13,1	7,8	6,5	10,4
Copagamento	2	2,0			2,0	1,4	2,5
Laboratório de imagem	4	45,4	13,7	77,0	38,9	23,3	60,9
Médico (outras)	19	30,4	15,7	45,2	14,8	11,7	37,6
Medicamentos	46	32,6	20,0	45,2	19,4	8,1	38,9
Suplementos	7	35,5	17,0	54,1	20,7	16,8	51,8
Transporte	20	9,5			1,9	1,2	4,2
Custos indiretos	66	127,8	53,1	202,5	47,9	20,0	123,2
Indiretos (familiar)	22	152,8	3,9	301,7	22,0	7,8	129,6
Indiretos (paciente)	56	90,6	54,6	126,6	47,9	22,3	110,2
Custo econômico	1.899	2.519	1.979	3.047	1.347	1.140	2.434

a) Intervalo interquartil.

Nota: As células vazias nos IC_95%_ não são significativas.

Na maioria dos grupos etários, os internados representaram a maior parte dos custos médicos totais, que aumentaram proporcionalmente com a idade. O acúmulo de custos totais concentrou-se em 53% dos indivíduos com 50 anos ou mais, grupo que acumula 27% dos casos (Figura 1A).

O modelo linear generalizado (Tabela 3) identificou que fatores como a idade (aumento adicional de 2% no custo por ano), ter uma comorbilidade (2,1 vezes) e ser sintomático (3,6 vezes) aumentam significativamente o custo médico direto médio pela covid-19, em comparação com aqueles indivíduos sem essas características. Esses fatores continuaram significativos para os custos hospitalares.

**Tabela 3:** ‒ Modelo de *ratios* de custos médicos diretos atribuídos à covid-19 e intervalo de confiança de 95% (IC_95%_), doentes de Córdoba, Colômbia, 2020 e 2021

Variáveis	Ratio de custos^a^	IC_95%_ inferior	IC_95%_ superior	p-valor	Efeitos marginais médios	IC_95%_ inferior	IC_95%_ superior	p-valor
Interceptação	23,56	17,45	31,81	< 0,001				
Sexo Masculino	1,05	0,87	1,28	0,601	26,96	-79,65	133,58	0,620
Idade (anos)	1,02	1,01	1,03	< 0,001	9,82	6,50	13,14	< 0,001
Regime de saúde Subsidiado	2,11	1,55	2,87	< 0,001	300,21	208,54	391,87	< 0,001
Caso da capital	1,62	1,32	1,99	< 0,001	245,42	135,14	355,70	< 0,001
Comorbilidade	2,05	1,57	2,69	< 0,001	457,88	191,13	724,64	0,001
Sintomático	3,56	2,87	4,42	< 0,001	456,77	369,90	543,65	< 0,001
N	1.800							
Pseudo R2 Nagelkerke	0,39							
Log Lik	-12 165,76							

a) Robust errors.

Por outro lado, o regime de afiliação subvencionada e o fato de o caso ter sido notificado em Montería implicaram aumento dos custos totais ambulatoriais e de internação domiciliar, mas não dos custos hospitalares.

Em termos de efeitos marginais, um ano adicional do paciente com covid-19 aumenta o custo do atendimento em US$ 9,8 (IC_95%_ 6,5;13,1). Além disso, a presença de pelo menos uma comorbidade implicou custo adicional médio de US$ 457,9 (IC_95%_ 191,1;724,6) em comparação com aqueles sem comorbidades (Tabela 3).

*Estimativa dos custos diretos não médicos e indiretos*

Na Tabela 4 são apresentadas as características sociodemográficas dos 99 casos de covid-19 entrevistados. Desses, 57,6% relataram despesas próprias relacionadas aos cuidados com a covid-19. O valor médio das despesas diretas pagas pelos pacientes e familiares foi de US$ 49,9 (IC_95%_ 33,6;66,2); dessas despesas, 55% foram em medicamentos (Tabela 2).

**Tabela 4:** ‒ Caracterização dos casos de covid-19 recuperados, entrevistas referentes às despesas diretas e à perda de produtividade, Córdoba, Colômbia, 2020 e 2021

Variável	Total (n = 99)
Pessoa que participou da entrevista	
Paciente	68 (68,7)
Cuidador	31 (31,3)
Dados socioeconômicos	
Idade, anos, média (DP)	42,9 (21,7)
Sexo	
Feminino	56 (56,6)
Masculino	43 (43,4)
Regime de saúde	
Subsidiado	83 (83,8)
Contributivo	16 (16,2)
Município de residência	
Montería	47 (47,5)
Lorica	16 (16,2)
Planeta Rica	5 (5,1)
Cereté	4 (4,0)
Chinú	3 (3,0)
Cotorra	3 (3,0)
Sahagún	3 (3,0)
Outros municípios	18 (18,2)
Faixa socioeconômica do domicílio	
Baixo-baixo (1)	76 (76,8)
Baixo (2)	22 (22,2)
Médio-baixo (3)	1 (1,0)
Escolaridade	
Ensino médio completo	25 (25,3)
Universidade (Superior)	17 (17,2)
Ensino fundamental completo	13 (13,1)
Técnico ou tecnológico	13 (13,1)
Ensino médio incompleto	12 (12,1)
Primário incompleto	11 (11,1)
Nenhum	7 (7,1)
Pós-graduação	1 (1,0)
Situação profissional e rendimento do familiar	
Ocupação antes do evento covid-19	
Trabalhava	55 (55,6)
Não trabalhava	16 (16,2)
Estudava	14 (14,1)
Trabalho doméstico	10 (10,1)
Outra	4 (4,0)
Trabalha atualmente	
Não	69 (69,7)
Sim	30 (30,3)
Motivo para estar desempregado	
Outro motivo	10 (41,7)
Mudança voluntária pela doença	7 (29,2)
Desligamento, não renovação do contrato	6 (25,0)
Pedido de membros da família	1 (4,2)
Número de pessoas na família, média (DP)	4,2 (1,7)
Rendimento do familiar	
Menos de 1 salário mínimo	75 (75,8)
1-2 salários mínimos	22 (22,2)
2-3 salários mínimos	2 (2,0)
Algum membro familiar recebeu subsídios	
Não	65 (65,7)
Sim	34 (34,3)
O rendimento do familiar cobre as despesas	
Não	89 (89,9)
Sim	10 (10,1)
Se considera pobre	
Sim	66 (66,7)
Não	33 (33,3)
Características em saúde	
Alguma comorbilidade	46 (46,5)
Fumante ou ex-fumante	17 (17,2)
Estado nutricional	
Normal	80 (80,8)
Sobrepeso	15 (15,2)
Obesidade	4 (4,0)
Atenção da covid-19^a^	
Ambulatório	90 (90,9)
Emergência	10 (10,1)
Hospitalar	8 (8,1)
Tempo da doença (dias), média (DP)	11,1 (13,4)
Tipo de caso	
Sintomático	53 (53,5)
Assintomático	46 (46,5)

a) Os doentes podem ter procurado serviços em mais de um contexto de cuidados, logo a soma dos doentes é superior ao total.

Por outro lado, 66,7% dos entrevistados referiram custos indiretos. O tempo médio despendido na covid-19 foi de 11,1 dias (DP = 13,4) e o tempo médio pelos familiares de 15,4 dias (DP = 21,6). Dos pacientes, 86,9% eram maiores de idade e 57,6% exerciam uma atividade remunerada com um rendimento médio mensal estimado em US$ 233,1 (DP = 125,6). O custo indireto por covid-19 foi estimado em US$ 127,8 (IC_95%_ 53,1;202,5) (Tabela 2).

A dimensão média dos familiares foi de 4,2 pessoas (DP = 1,7), com uma renda mensal média de US$ 204,4 (DP = 121,3), representando em média 16,5% da renda familiar. Em termos de quintis de rendimento, essa proporção aumentou para os familiares com rendimentos mais baixos, atingindo 21,7% (Figura 1B). Os custos indiretos refletem a perda estimada de rendimento das famílias devido à doença, representando 54,2% do rendimento; uma proporção que, no quintil mais baixo, representava 101% do rendimento total das famílias (Figura 1C).

**Figura 1:** ‒ Custos diretos médicos por contexto de atenção e idade, despesas diretas e custos indiretos em relação ao rendimento do familiar, por quintil de rendimento, atribuíveis à covid-19 no departamento de Córdoba, Colômbia, 2020 e 2021

Nota: As informações relativas a custos diretos médicos referem-se ao período março-setembro de 2020. As relativas a despesas próprias e custos indiretos referem-se ao período de janeiro-fevereiro 2021.

Os familiares recorreram a diferentes fontes de financiamento para cobrir as despesas incorridas com a covid-19. Desses familiares, 39,4% utilizaram poupanças, 35,4% outras fontes, 27,3% solicitaram dinheiro emprestado e 10,1% necessitaram de donativos. No quintil de renda mais baixo, a utilização de poupanças foi a maior fonte de financiamento, com 45%.

*Custo econômico estimado*

O custo econômico médio estimado da covid-19 no departamento de Córdoba foi de US$ 2.519 (IC_95%_ 1.980;3.047), 92,9% composto por custos médicos diretos. Por sua vez, o custo econômico mediano foi de US$ 1.347 (IIQ 1.140;2.434) (Tabela 2). Em 14 de abril de 2020, o Instituto Nacional de Saúde comunicou um total de 46.233 casos confirmados de covid-19 no departamento de Córdoba;^2^ então, o custo econômico extrapolado para o total dos casos estaria entre US$ 116,5 e US$ 118,1 milhões.

## DISCUSSÃO

Este estudo estimou um custo econômico médio da covid-19 no departamento de Córdoba por episódio de US$ 2.519 (IC_95%_ 1.979;3.047), 92,9% para custos médicos diretos; as despesas diretas e indiretas representarem 2% e 5,1%, respectivamente. Com base nesse custo, estimou-se que a carga econômica para o departamento representava entre 2,4% e 2,5% do produto interno bruto (PIB) do departamento para 2019 (US$ 4.741 milhões).^18^ Em termos *per capita* (US$ 2.622), o custo econômico de um evento médio de covid-19 em Córdoba representaria entre 75,5% e 116,2%.

As despesas diretas estimadas neste estudo representam, em média, 16,5% da renda do familiar, com um maior peso no quintil de renda mais baixo (21,7%). Por sua vez, os custos indiretos refletem a perda estimada do rendimento do familiar decorrente da doença, significando 54,2% da renda. De acordo com a medida de carência monetária (2019), Córdoba era um dos três departamentos com a faixa de pobreza mais baixa do país (US$ 67,8) e a segunda faixa de pobreza extrema mais baixa (US$ 31,5), evidenciada pela porcentagem da população com uma renda *per capita* da unidade de despesa abaixo desses limites (54,2% e 15,2%, respetivamente).^19^ As despesas diretas estimadas neste estudo indicariam que 277 mil pessoas no departamento não seriam capazes de cobrir o custo da doença com os seus rendimentos (com a população de 2020). Em termos de perda de produtividade, 991 mil pessoas no departamento veriam potencialmente os seus rendimentos e os dos seus familiares sendo afetados pela covid-19.

A perda de rendimentos e a carga para os mais vulneráveis diante da covid-19 apresentam uma dimensão maior se consideramos a perda de emprego devido às restrições em todos os setores econômicos. Durante 2020, Córdoba possuía uma taxa de desemprego de 15,7%, e Montería uma taxa de informalidade de 59,9%.^20^ A este respeito, Bonnet et al.^21^ e Ricciulli-Marín et al.^22^ estimaram o impacto regional da covid-19, mostrando como as restrições à mobilidade em Córdoba implicariam perdas de 5,7% do PIB, principalmente nos setores de construção civil e demais serviços. De fato, neste estudo, 49,1% das pessoas que afirmaram trabalhar antes de contrair a doença declararam que não trabalhavam, no momento da entrevista. Destes últimos, 25% indicaram que a causa foi o desligamento ou a não renovação do contrato de trabalho e outros 29,2% afirmaram não trabalhar mais devido à doença.

A respeito do custo médico direto médio, este é, em grande parte, explicado pela hospitalização, em que os casos com internação hospitalar tiveram custos 16,2 vezes mais elevados do que os menos graves. Nossa estimativa foi semelhante aos US$ 2.283 (IIQ 788;2.523) calculados por Alvis-Zakzuk et al. para um caso médio na Colômbia.^23^ Outras, como a de Bartsch et al.,^24^ indicaram um custo médico direto médio de US$ 3.045. Já Li et al.,^25^ através de microcusteio, descobriram que um paciente custava em média US$ 6.827 por evento, com o custo concentrado nos medicamentos (45% do custo médio). A respeito dos custos hospitalares, nossos resultados indicam um custo médio de hospitalização geral de US$ 1.078 (IC_95%_ 942,8;1.213) e um custo médio de UTI de US$ 4.076 (IC_95%_ 3.520;4.632). Resultados semelhantes foram publicados por Khan et al.,^26^ cujo custo médio de hospitalização por paciente foi de US$ 1.385 (DP = 166,04).

O custo médico direto médio varia de acordo com fatores como idade, comorbilidades e sintomatologia. Observou-se que o regime de afiliação e a notificação do caso na capital aumentariam o custo total e os custos ambulatoriais e domiciliares, mas não os custos hospitalares. Esse resultado mostra as condições de acesso aos serviços de cuidados domiciliares e ambulatoriais na capital em comparação com os outros municípios, juntamente com uma concentração de casos na capital (47,3% em Montería). Uma vez que as despesas médicas diretas foram recolhidas numa seguradora em que o regime subsidiado é majoritário, os resultados refletem o desequilíbrio da amostra entre os dois regimes de inscrição e a singularidade da seguradora.

Há, no estudo, limitações importantes a se considerar. Em primeiro lugar, os resultados estimados são parciais, dado o período do estudo. Em segundo lugar, as bases de dados disponíveis se apresentavam incompletas em alguns registros, o que poderia subestimar os custos relacionados com medicamentos no componente de custos médicos diretos. Essa limitação poderia ser mitigada com o alargamento da amostra de casos de covid-19 em outras seguradoras, reduzindo-se os vieses de estimativa e aumentando a precisão dos resultados. Uma terceira limitação é o fato de os custos econômicos estimados não terem levado em conta os custos associados à perda de horas de trabalho ou aos desligamentos relacionados com as medidas tomadas pela população para limitar a transmissão do vírus, durante a pandemia (isolamento preventivo obrigatório), e o tempo despendido pelos prestadores de cuidados ou pelos familiares que cuidaram dos doentes. De fato, essas medidas populacionais geraram perdas econômicas estimadas entre US$ 1,2 milhão e US$ 15,3 milhões por mês de isolamento, equivalentes a 0,5% a 6,1% do PIB nacional.^21^ Deve-se ressaltar que o presente estudo não apresenta custos da covid-19 longa. É importante, em análises futuras, estimar os custos subsequentes causados pela incapacidade gerada pela covid-19, a fim de completar a estimativa da carga econômica dessa doença. Outra limitação é o potencial viés de memória ao se relatarem custos, por meio de ligação telefônica, em períodos do passado. Perguntar por despesas do passado poderia subestimar as estimativas de despesas próprias. Entretanto, um período de sete dias não é tão longo a ponto de ser significativo.^27^

Dado o impacto econômico significativo da covid-19, especialmente nas famílias de baixa renda e no aumento da perda de empregos, as políticas de saúde devem se concentrar em duas áreas cruciais. Primeiro, é necessário dar atenção específica à proteção financeira das famílias vulneráveis por meio de programas de apoio financeiro. Segundo, é necessário implementar medidas para preservar o emprego e a renda, como apoio ao emprego e subsídios comerciais. A interconexão entre a saúde física e a econômica destaca a importância de uma abordagem abrangente da saúde pública, abordando-se não apenas a prevenção e o tratamento de doenças, mas também seus impactos sociais e econômicos, com medidas para atenuar as desigualdades. 

Estimar os custos de uma doença é uma necessidade para qualquer sistema de saúde, especialmente aqueles relacionados a despesas próprias e custos indiretos relacionados à perda de produtividade. Esses são os principais insumos para avaliar o custo-efetividade de novas intervenções de saúde para ajudar a mitigar os efeitos da doença. Nesse sentido, os métodos usados neste artigo podem ser replicados em outros sistemas de saúde e em outras latitudes. Análises futuras podem avaliar os custos da doença para a covid-19, covid longa e doenças respiratórias relacionadas, levando em consideração a natureza do sistema de saúde (privado e público), a variabilidade geográfica e as desigualdades socioeconômicas da população.

Este estudo demonstrou a elevada carga econômica que o sistema de saúde e as famílias do departamento de Córdoba enfrentaram devido à covid-19. As famílias mais vulneráveis precisaram arcar com uma grande parte da carga sobre os seus rendimentos, com consequências importantes para o progresso social e a superação da pobreza extrema.

## Figures and Tables

**Figura Suplementar 1:** ‒ Processo de seleção amostral para estimativa das despesas próprias e custos indiretos, Córdoba, Colômbia, 2020 e 2021
